# Revised Age Estimates for Northern Resident Killer Whales (*Orcinus orca*) Based on Observed Life‐History Events and Demographic Discounting

**DOI:** 10.1002/ece3.70981

**Published:** 2025-03-09

**Authors:** Andrew W. Bateman, Jessica MacLean, Eva Stredulinsky, Brianna Wright, Graeme Ellis, Thomas Doniol‐Valcroze, Chris Darimont, John K. B. Ford

**Affiliations:** ^1^ Department of Geography University of Victoria Victoria British Columbia Canada; ^2^ Department of Ecology and Evolutionary Biology University of Toronto Toronto Ontario Canada; ^3^ Fisheries and Oceans Canada Pacific Biological Station Nanaimo British Columbia Canada

## Abstract

Long‐term field studies have been invaluable in the study of ecology and evolution; however, for particularly long‐lived species, even long‐term studies often rely on estimated ages, for example when investigating demographic processes. One approach is to estimate unknown birth dates from the known timing of other life‐history events. Building on previous methods, we update estimation techniques for Northern Resident killer whales (NRKW; *Orcinus orca* ) as part of an ongoing long‐term study that began in 1973. Despite almost 50 years of observation, many individuals were born before records began, and detailed understanding of NRKW life history relies on estimated ages. Our age estimation approach incorporates new data from photo‐identification surveys into a framework that relies on accrued knowledge of demographic rates from known‐age individuals. We use Bayes' law to determine conditional probability distributions from age‐at‐event data, incorporating mathematical descriptions of demographic patterns parameterised from the data. Key to our approach is the discounting of higher age estimates due to the increasing likelihood of mortality with age, a pattern not previously taken into account for NRKWs. We estimate ages for multiple age and sex classes of individuals, using related but tailored approaches, and we incorporate uncertainty into our estimates. Our revised age estimates suggest that individuals are often younger than previously thought (3.5 years on average across 73 individuals; range: 0–15 years). Moreover, the largest discrepancies appear for mothers with offspring at the onset of the study, a class of individuals instrumental for investigating menopause in killer whales—one of the few species other than humans known to exhibit this life‐history feature. Our results will ultimately enable a refined understanding of the evolutionary forces that produce such patterns. We discuss the implications of our findings for the study of resident killer whales and for age estimation in other long‐lived animals.

## Graphical abstract

Even some long‐term studies must rely on estimated ages when particularly long‐lived species are involved. Using empirical probabilities of life‐history event timing, we update age estimates for Northern Resident killer whales that were born before the start of observation, five decades ago. A feature of our methods novel to this system—discounting higher age estimates to reflect accrued mortality with age—means that many individuals appear to be younger (grey shading) than previously thought (red shading). Distributions shown represent age estimates for a grandmother and her daughter and grandson.

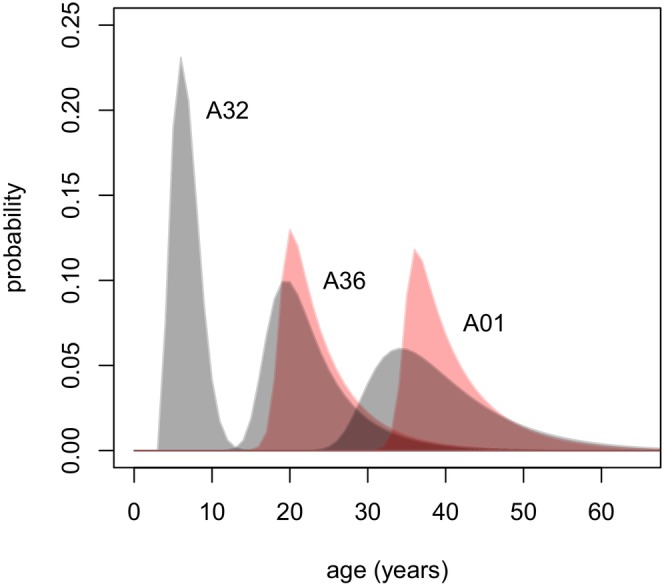

## 1 Introduction

Long‐term ecological field research, spanning decades, has advanced our understanding of ecological and evolutionary processes by tracking the development, behaviours and life histories of known individuals within animal populations (Clutton‐Brock and Sheldon 2010; Lindenmayer et al. 2012; Hayes and Schradin 2017; Taig‐Johnston et al. 2017). For many long‐term research programs, accurately estimating the timing of life‐history events—particularly birth and death—is critical to inference and insight. Demographic data, such as individual age, provide the foundation to examine biological processes at multiple scales (Clutton‐Brock and Sheldon 2010). For example, knowledge of individual ages has been necessary to infer post‐reproductive lifespans in Asian elephants (*Elaphus maximus*; Chapman et al. 2019) and killer whales ( *Orcinus orca* ; Olesiuk et al. 1990; Franks et al. 2016), as well as generate evidence that post‐reproductive females can increase the fitness of their descendants (the “grandmother effect”; Foster et al. 2012; Brent et al. 2015; Lahdenperä et al. 2016; Nattrass et al. 2019). Whereas estimating the time of death usually requires observing or inferring a recent event, estimating birth timing, and therefore age, can pose particular challenges in long‐lived animals. Populations of long‐lived animals, such as elephants, cetaceans and even rockfish (*Sebastes* spp.), contain individuals for which births can predate the start of multi‐decadal studies (Cailliet et al. 2001; Fritz 2017; Mann and Karniski 2017). For such long‐lived species, we must rely on alternative methods to estimate ages.

In the case of resident killer whales (RKW), salmon‐specialist predators that inhabit the northeast Pacific Ocean (Ford et al. 1998, 2010), discovery of individually identifiable natural markings in the early 1970s (Bigg 1982) enabled researchers to track individual fates. More than four decades of detailed study across multiple populations of these apex predators has ensued (Matkin, Ellis, et al. 1999; Matkin, Saulitis, et al. 1999; Vélez‐Espino et al. 2014; Center for Whale Research 2016; Towers et al. 2020). RKWs comprise a genetically distinct killer whale ecotype (Morin et al. 2010) and are further divided into genetically and socially distinct populations (Ford 1991; Hoelzel et al. 2007). Northern and southern populations (NRKWs and SRKWs, respectively) collectively range from California to southeast Alaska, with range overlap in British Columbia and Washington. Due to their small population sizes and, in the case of SRKWs, declining population trajectory, these populations have long been subjects of conservation concern (COSEWIC 2008). NRKWs are listed as Threatened and SRKWs as Endangered under Canada's *Species At Risk Act* (Fisheries and Oceans Canada 2011), and SRKWs are listed as Endangered under the United States' *Endangered Species Act* (National Marine Fisheries Service 2021).

Multiple research programs have produced insights into killer whale ecology and life history, including the discovery of group‐specific acoustic dialects (Ford 1991; Yurk et al. 2002), social mechanisms of philopatry (Wright et al. 2016) and reproductive senescence (Foster et al. 2012; Brent et al. 2015; Nielsen et al. 2021). At times, these studies have also led to broader evolutionary insights (e.g., Croft et al. 2017). Findings related to matrilineal social structure, long lifespans and reproductive senescence of RKWs have also offered new insight into the role of population dynamics and behaviour in the context of conservation (Franks et al. 2016; Wright et al. 2016; Nattrass et al. 2019; Stredulinsky et al. 2021). In many cases, age estimates comprised important inputs for such studies of demography and behaviour.

First censused in 1973, NRKWs have been studied using annual photographic identification surveys (Bigg 1982; Olesiuk et al. 2005; Towers et al. 2020; DFO 2022), allowing for a near‐comprehensive individual‐based dataset spanning 50 years. Despite the duration of the NRKW study, knowledge gaps remain regarding demographic and social processes, in part due to female lifespans exceeding the length of study (Olesiuk et al. 2005; Brent et al. 2015). As a result, demographic rates, age effects and complex life‐history findings have all relied on estimated ages for older individuals that matured prior to the 1970s.

Past estimates of NRKW ages relied on a number of techniques. For young calves, researchers were able to directly estimate ages with low uncertainty, based on clear growth patterns (Olesiuk et al. 1990). For older individuals, researchers inferred unknown ages for NRKWs born before the start of the photographic census by using timings of life‐history events from known‐age individuals to estimate unknown birth years in relation to those events (Bigg et al. 1990; Olesiuk et al. 1990, 2005). These ‘demographic’ age estimation techniques have been employed in multiple studies of NRKW biology, applied to SRKWs in parallel, used to understand conservation challenges faced by RKW populations (Foster et al. 2012; Vélez‐Espino et al. 2014; Brent et al. 2015; Wright et al. 2016; Croft et al. 2017; Nattrass et al. 2019; Murray et al. 2021), and recently used to validate molecular age estimation techniques (Parsons et al. 2023). Although the existing age estimates for older individuals did incorporate decades of demographic data, they did not incorporate an important demographic reality: all else being equal, individuals born earlier are less likely to be observed because they are more likely to have died.

Building on past work, we updated the demographic age estimation techniques to incorporate new data from recent photo identification surveys of the NRKW population and to discount older ages based on estimated mortality rates. Our analyses draw on Bayes' theorem to provide age estimates, offering a transparent methodology that could be adapted to other systems. Our findings provide more accurate (less biased) age estimates than those previously available for NRKWs and, as a result, can help refine insight into critical biological problems and issues of conservation concern.

## 2 Methods and Results

### 2.1 Data Summary

Since 1973, researchers have performed an annual photographic census of the NRKW population. Individuals are identified via unique scarring and colouration patterns on their dorsal fins and saddle patches, and maternal relationships are determined via repeated observations of close social associations—an approach validated by genetic analyses (Barrett‐Lennard 2000). Concerted effort is made to photograph each individual at least once every year, facilitated by NRKW's highly social and strongly philopatric behaviour. Photographic census data are then used to assign age and life‐history status to individuals, thus generating a conventional demographic census methods described elsewhere; (DFO 2022). The analyses described in this paper use census data collected through the end of 2016.

### 2.2 Analytical Approach

We built on the methods of past authors (Bigg et al. 1990; Olesiuk et al. 1990, 2005), who estimated age ‘correction factors’ for Northern Resident killer whales first observed as adults at the start of the photo‐identification census in 1973. Detailed methods have been described in the previous reports, but briefly, the methods derive age estimates for unknown‐age individuals using the relative frequencies of key life‐history events occurring at specific ages in known‐age individuals. For females, the key life‐history event used for ageing is the birth of a female's first known calf, accounting for elevated early life offspring mortality (Olesiuk et al. 1990). For males, the key events are maturation transitions, as determined by the changing morphology of a male's dorsal fin with sexual and physical maturity (Olesiuk et al. 1990). Our approach mirrors that of the previous authors, but additionally incorporates a key demographic reality that had not been accounted for in prior age estimation methodologies: all else being equal, older individuals are less likely to be observed because they are more likely to have died than younger individuals.

Our age estimation methods rely on mathematical descriptions of NRKW demographic rates, which we chose to approximate previously described patterns (e.g., Ward et al. 2009; Ford et al. 2010) and fit to observed data. We performed all analyses in R, fitting models to data using the *optim* algorithm (R Development Core Team 2021) to minimise the appropriate negative log likelihood functions (e.g., binomial negative log likelihood in the case of annual survival and reproduction probabilities).

We begin here by describing methods for females first observed with offspring, and we then elaborate methods for other classes of individuals.

We estimated the conditional probability of a female's age, given the age of her oldest living offspring:

(1) $$P\left({\text{mother}}^{'}s\;\mathrm{age}=x|\text{oldest living offspring}\;\mathrm{age}=y\right)$$

written for convenience as P(*x*|*y*). This approach employed Bayes' theorem to incorporate the conditional probability distribution of oldest‐living‐offspring age, given female age, P(*y*|*x*); the probability distribution of living females' ages in the population, P(*x*); and the probability distribution of the age of a female's oldest living offspring, P(*y*):

(2) $$P\left(x|y\right)=\frac{P\left(y|x\right)P\left(x\right)}{P\left(y\right)}$$

Each component on the right‐hand side of Equation (2) is detailed below. An important feature of the conditional probability distribution (1) is that it depends on the population growth rate, which affects population age structure and thereby P(*x*).

For all calculations, we assumed that individuals are age zero at their first census after birth. Thus, an individual of age *t* has been censused *t* + 1 times.

*Estimation of* P(*y|x*).

For a *x*‐year‐old female's oldest living offspring to be *y* years old, she would have to have no surviving offspring born before she was age *x* − *y*, and then have given birth at age *x* − *y* to an offspring that survived for *y* years.

Given offspring survivorship to *t, S*(*t*), and the probability of a female reproducing at age *t* (fecundity), *R*(*t*), the probability that a female of age *x* had an offspring at age *t* that survived to be *x* − *t* is *R*(*t*)*S*(*x* − *t*), and

(3) $$P\left(y|x\right)=\left[\prod_{t=0}^{x-y-1}\left[1-R\left(t\right)S\left(x-t\right)\right]\right]R\left(x-y\right)S\left(y\right)$$

**FIGURE 1:**
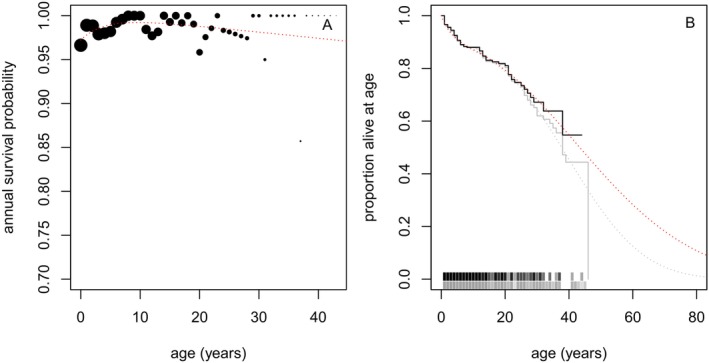
Annual survival (A) observed (points, area proportional to sample size) and estimated (red dashed curve), and survivorship (B) observed (black) and estimated (red dashed) for NRKWs of known age with surviving mothers. Grey curves in (B) indicate observed (solid) and estimated (dashed) survivorship for all individuals of known age. “Rugs” above the age axis in (B) indicate censoring in survival data (i.e., ages that surviving individuals were known to achieve in the ongoing study), with colours corresponding to the empirical survivorship curves.

To estimate survivorship to age *t*, accounting for known elevated early life mortality (Olesiuk et al. 1990), we assumed a ‘bathtub shaped’ hazard function, *h*(*t*), consisting of a standard Weibull hazard component plus an exponentially decaying early life hazard component. This took the form: *h*(*t*) = *a*·exp.(*−bt*) + (c*/d*)(*t/d*)^*c −* 1^, with free parameters *a*, *b*, *c* and *d* to be estimated. Using standard calculations, survivorship *S*(*t*) is equal to exp.($-\int_{0}^{t}h\left(\tau \right)\mathrm{d\tau}$) and annual survival probability, conditional on survival to age *t*, is *S*(*t* + 1)*/S*(*t*). That is, annual survival from age *t* is exp($-\int_{t}^{t+1}h\left(\tau \right)\mathrm{d\tau}$), where *τ* is a time variable used solely for integration. We estimated parameters in the hazard function by fitting this annual survival function to observed annual survival data for NRKWs with surviving mothers (Figure 1), and we then used those parameters to calculate *S*(*t*).

**FIGURE 2:**
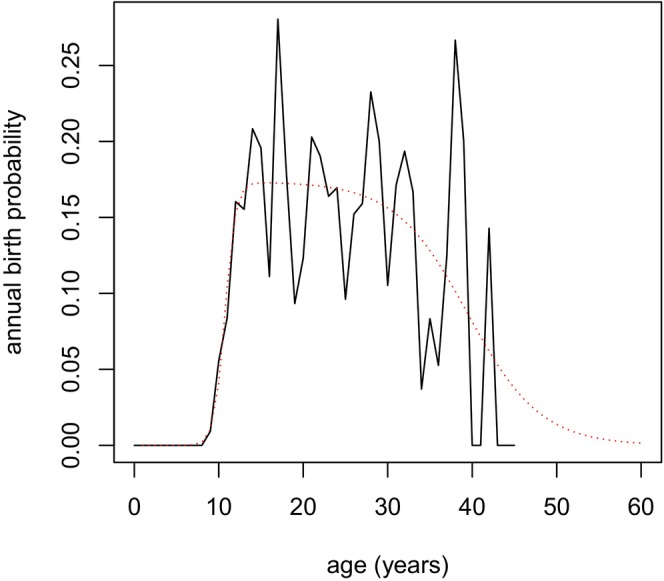
Annual observed (black) and estimated (red dashed) probability of producing a viable calf for surviving NRKW females.

Given that NRKW female fecundity increases rapidly at maturity, around age 10 years, but undergoes an accelerating decline after about age 25 years (Ward et al. 2009), we described age‐specific fecundity, *R*(*t*), using two logistic functions, one constrained to be increasing and the other constrained to be decreasing. The product of the logistic functions produced the requisite ‘hump‐shaped’ curve, which we scaled by a theoretical maximum annual reproduction probability (Figure 2). Specifically, we used *R*(*t*) = *R*_max_·(1 + exp.[−*α*_0_ − *αt*])^−1^·(1 + exp.[*−β*_0_ − *βt*])^−1^, where *R*_max_, *α*_0_ (< 0), *α* (> 0), *β*_0_ (> 0), and *β* (< 0) were free parameters, estimated using reproduction data for known‐age females.

**FIGURE 3:**
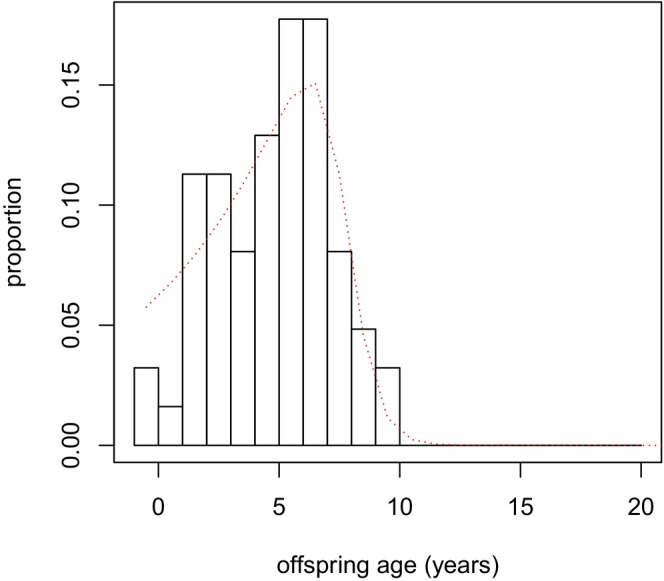
Observed (bars) and estimated (red dashed curve) distribution of age of oldest living offspring for 20‐year‐old NRKW females. Estimates generated from a mathematical model informed by NRKW demographic data.

As an example, Figure 3 shows P(*y*|20), the distribution of ages for the oldest living offspring of female NRKWs known to be 20 years old.

### 2.3 Estimation of P(*x*)

**FIGURE 4:**
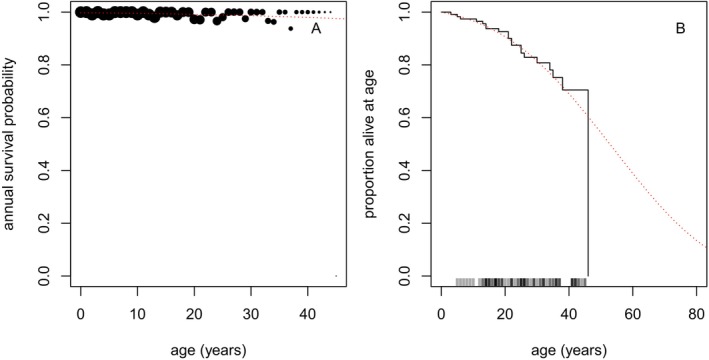
Annual survival (A) observed (points, area proportional to sample size) and estimated (red dashed curve), and survivorship (B) observed (black) and estimated (red dashed) for female NRKWs of known age. “Rug” above the age axis in (B) indicates censoring in survival data (i.e., ages that surviving individuals were known to achieve in the ongoing study) corresponding to the empirical survivorship curve. Note lack of early life mortality in B, relative to Figure 1B, due to survivorship estimation bias (individuals that die before being sexed are absent from the analysis).

P(*x*) is simply the age distribution of females. For a stable population, this would be the female survivorship function, *S*_*f*_(*t*). Because the estimation of a mother's age is inherently dependent on her being a female, we estimated annual survival for females of known age (Figure 4A), in order to estimate *S*_*f*_(*t*) (Figure 4B) similarly to *S*(*t*), above.

Estimating survival for individuals known to be female excluded females that died before they could be sexed. If left unaddressed when estimating P(*x*), this ‘survivorship bias’ would artificially inflate the estimated survival of young individuals and result in inappropriate discounting. To address this bias, we used the hazard estimate for all individuals of known age to impute the female‐only hazard function estimate before age 15, although this had minimal effect on the final age estimates.

**FIGURE 5:**
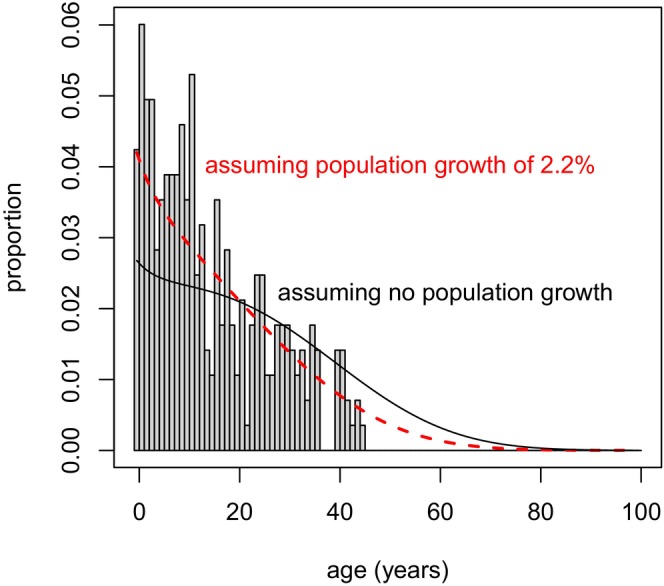
Observed (bars) and estimated (red dashed curve) age distribution for NRKWs of known age in 2015, assuming an annual population growth rate of 2.2% (𝜆 = 1.022). The black curve shows the estimated age distribution assuming a stable population. Note that the empirical age distribution is truncated due to the limited (≈40 year) data set used in this study.

For a population growing at annual rate 𝜆, each year sees an average of 𝜆 times as many female offspring produced as in the previous year, so younger age classes are more prevalent than in a stable population, and P(*x*) must be adjusted to take population growth into account. In this case, P(*x*) becomes the normalised version of function *f*(*t*) = *S*_*f*_(*t*)𝜆^−*t*^. In generating P(*x*), we used the average historical (1973–2016) annual population growth rate of 𝜆 = 1.022. To check the effect of this assumption with the most data possible, we generated an estimated age distribution for all individuals, P_all_(*x*), calculated in the same way as for the female‐only portion of the population. This estimate was consistent with the observed age distribution in 2015 (Figure 5).

### 2.4 Estimation of P(*y*)

We estimated P(*y*) by summing across its partition with respect to maternal age, *x*:

(4) $$P\left(y\right)=\sum_{x=0}^{\infty }P\left(y|x\right)P\left(x\right)$$

In practice, we truncated the summation at *x* = 100, by which time most females have died (Figure 4B).

### 2.5 Final Form of P(*x*|*y*)

**FIGURE 6:**
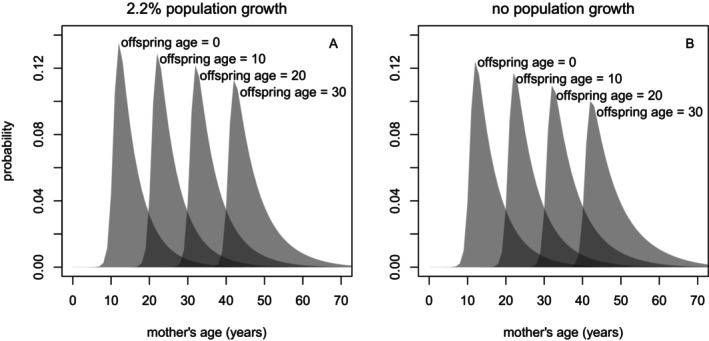
Probability distributions for a mother's age, given her oldest living offspring's age, for NRKWs at population growth rates of 2.2% (𝜆 = 1.022; A) and 0% (B).

Bringing together the component parts, we arrived at the final empirically informed distribution of maternal age, given the age of her oldest living offspring (examples shown in Figure 6). Relative to P(*x*|*y*) distributions calculated assuming 2.2% annual population growth, P(*x*|*y*) distributions assuming no population growth showed slightly more uncertainty (i.e., a slightly ‘wider’ distribution of possible maternal age for a given offspring age).

### 2.6 Age Estimates From Other Life‐History Events

In addition to age estimates based on mothers' oldest living offspring, we estimated ages from the timing of other life‐history events, using the same set of events and general conceptual approach used by Olesiuk et al. (1990), with the methodological addition of age‐based discounting to account for declining survivorship, as described above. For females without offspring at the onset of detailed study, we derived estimates using the birth timing of their first known offspring. For males, we derived estimates based on the timing of maturation events, as determined by changing morphology of their dorsal fin (Olesiuk et al. 1990). These events, in declining order of preference, were: onset of dorsal fin growth (“sprouting”), attainment of a full‐sized dorsal fin, and initial observation with a full‐sized dorsal fin (for males already mature at the start of the study). The associated methods elaborate on, or parallel, those described above, and we reserve details for the Appendix S1. Where possible, we shared information (models) across these analyses. For example, we estimated the overall survivorship relationship for females of known age (*S*_*f*_(*t*); Figure 4) only once and used it across all relevant calculations (i.e., Equations (2) and (S1), in this case).

### 2.7 Accounting for Uncertainty in Offspring Age

For females first observed with offspring at the start of the study, the age of those offspring had to be estimated using one of the methods outlined above, or, for very young individuals, by direct estimation based on size and morphology (Olesiuk et al. 1990). Thus, the age of offspring used to estimate a mother's age, via Equation (2), could itself be uncertain. We take this to imply a probability associated with each possible offspring age, *y*, given the estimated offspring age, *y*_*e*_, denoted P(*y* | *y*_*e*_). The distribution of a mother's age, given her offspring's estimated age, becomes:

(5) $$P\left(x|y_{e}\right)=\sum_{y}P\left(x|y\right)P\left(y|y_{e}\right)$$

**FIGURE 7:**
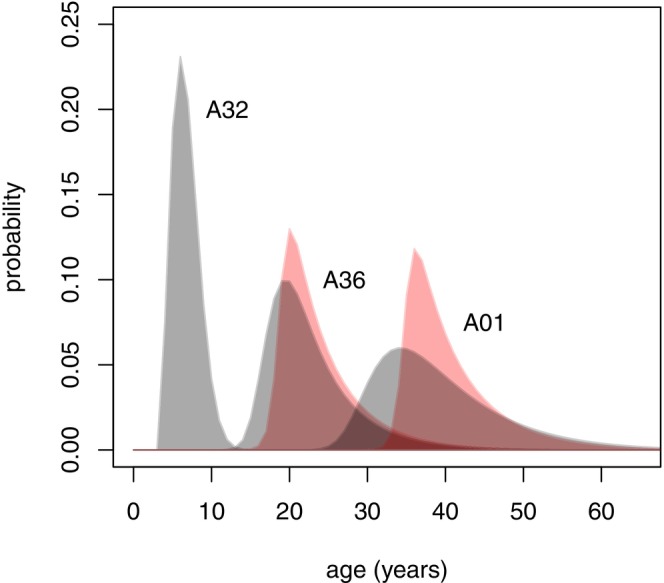
Probability distributions for NRKW ages, spanning three generations. Left, centre, and right distributions show age‐estimate uncertainty for a male (A32), his mother (A36), and her mother (A01), respectively, at the start of the NRKW study. Red distributions are based on original point estimates of offspring age (Olesiuk et al. 2005); grey distributions incorporate uncertainty about offspring age. Distributions assume a NRKW population growth rate of 2.2%. Uncertainty associated with inference of relationships is not reflected in these estimates.

For some females, we estimated age based on a daughter's age, which we in turn estimated based on the age of that daughter's son (Figure 7). This required application of Equation (5) twice, starting with estimation of the daughter's age. Given the assignment of relationships in this scenario, inferred via behavioural interactions that were necessarily first observed when multiple individuals involved were already sexually mature, we note that there is additional inherent uncertainty not captured by the probability distributions we present.

When offspring age had previously been estimated directly, based on size and morphology, we used the estimated (Olesiuk et al. 1990) mean and reported uncertainty to parameterise a gamma distribution for P(*y*|*y*_*e*_), with mean equal to the estimated offspring age and standard deviation equal to half the estimated uncertainty in offspring age.

### 2.8 Final Estimates

**FIGURE 8:**
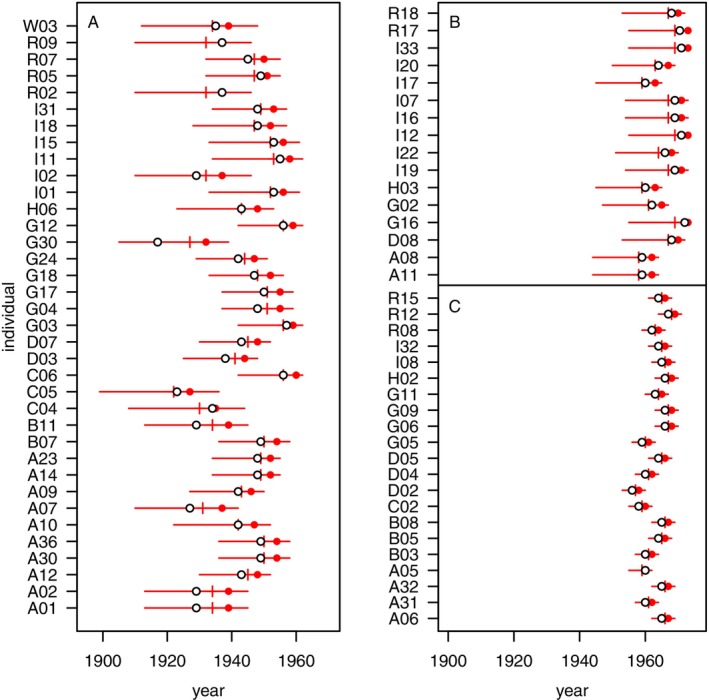
Existing (open circles) and revised modal (red circles), mean (vertical segments), and 2.5–97.5 percentile range (horizontal segments) year‐of‐birth estimates for NRKW (A) females first seen with offspring, (B) females of unknown age when first seen that later gave birth to a calf, and (C) males of unknown age and immature when first seen.

Using the methods described above and in the Appendix, we calculated age distributions for 96 individuals of unknown ages at the onset of the NRKW study, and we used these to calculate associated birth‐year distributions (Figure S5). The estimated age distributions are strongly peaked, with a long tail skewed towards earlier birth years (Figure 8 and Figure S5), and birth‐year distributions show a wide range between 2.5th and 97.5th percentiles, especially for females (Figure 8). Other than for 23 males that were first observed mature and previously received only estimates of minimum age (Olesiuk et al. 1990), our revised modal (most probable) birth‐year estimates are generally more recent than previous estimates (Figure 8). Relative to the corresponding previously reported age estimates, our revised modal estimates decreased by an average of 5.0 years (range: 0–15 years; *n* = 36) for females first observed with an inferred offspring, 2.3 years (1–3 years; *n* = 16) for females that first gave birth during the study, and 1.9 years (0–2 years; *n* = 21) for males that were immature at the start of the study. Across these 73 individuals our age estimates are, on average, 3.5 years lower. This reflects the fact that older individuals are, overall, less likely to be observed than younger individuals in a stable or growing population, as a result of survival monotonically decreasing with age. For males that were physically mature at the start of the study, our age estimates are an average of 2.0 years (1–4 years; *n* = 23) higher than the previous minimum‐age estimates. We provide mean, median and modal values for the birth year distributions calculated in this study (Table S1), and we provide the associated birth‐year probability distributions in the Supporting Information. We further provide least‐squares parameter estimates for lognormal distributions fit to the empirical distributions we calculated (Table S2: see supplemental code).

Age estimates for several individuals used the techniques described here, but drew on data collected outside of the main long‐term study. Three mothers (A14, A23 and C05) had offspring (A17, A16 and C11, respectively) that had been captured for the aquarium trade prior to the start of the study. The age estimates for these three mothers reflect the estimated ages of these captured offspring. Also, male I10 was aged based on identification in a photograph, taken in 1968 when he had already achieved asymptotic dorsal fin size.

**FIGURE 9:**
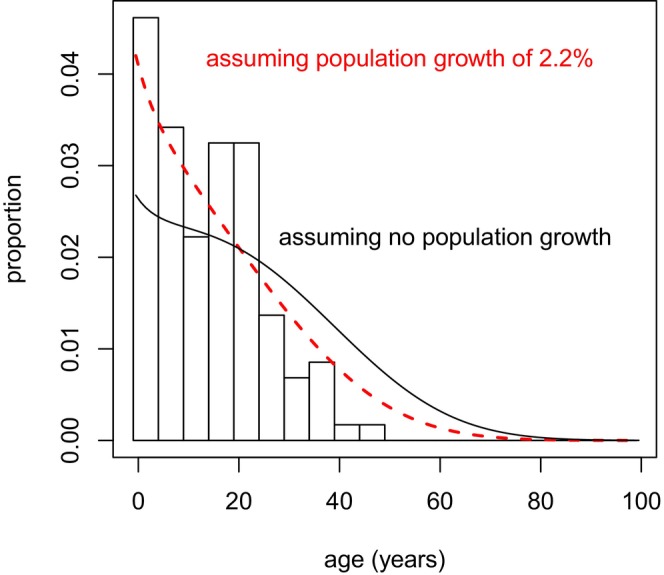
Age distribution for NRKWs at the start of detailed study, in 1973. Empirical distribution shows revised age estimates (bars) compared to theoretical age distribution (red dashed curve), assuming an annual population growth rate of 2.2% (𝜆 = 1.022). The black curve shows the estimated age distribution assuming a stable population.

As a final validation of our methods, we collated our revised age estimates for individuals present at the start of the NRKW study in 1973, and compared them to the theoretical stable age distributions assuming 0% and 2.2% annual population growth (Figure 9). The theoretical distribution corresponding to 2.2% growth matched well with the empirical distribution of age estimates, generally corroborating our assumption of 2.2% population growth for the years before the study began (but note that the population had been impacted by removal of some individuals for the aquarium trade).

## 3 Discussion

Using photo‐identification data collected since the early 1970s, and building on previously described methods (Bigg et al. 1990; Olesiuk et al. 1990, 2005), we generated age estimates for northern resident killer whales (NRKW) with unknown birth years. Our age estimation methods drew on information about the timing of key life‐history events, including age of first reproduction in females and age of maturation in males, and integrated information about survivorship of focal individuals and their offspring. For females, we based ages on the timing of the birth of their oldest surviving calves. For males, we based ages on the timing of characteristic stages of dorsal fin growth, which had been documented for past ageing work (Bigg et al. 1990; Olesiuk et al. 1990, 2005). Whereas our methods generally paralleled past approaches to age estimation in NRKWs (Bigg et al. 1990; Olesiuk et al. 1990, 2005), we incorporated one key improvement: the need to discount older age estimates to account for natural mortality. This discounting resulted naturally from our use of Baye's formula to estimate ages. In any stable or growing population exhibiting stable age structure, older individuals are less common than younger individuals, since the number of individuals alive at older ages declines due to mortality. By appropriately discounting older ages, we provide more realistic age estimates than previously available for NRKWs.

Our results (Figure 8) suggest that a number of NRKWs born before the start of the detailed study are likely years younger than previously reported (Bigg et al. 1990; Olesiuk et al. 1990), with point estimates for several individuals more than 10 years lower than previous estimates. Revisions to age point estimates were particularly apparent for NRKW females that were first seen with offspring at the start of the study (e.g., individuals A01, A02, B11, G30; Figure 8 and Table S1). In the extreme, female G30, first seen with an offspring, appears to have been almost 15 years younger than previously reported. We caution, however, that age estimates for these four noted females are based on inferred—and particularly uncertain—mother‐offspring relationships, and the females died before genetic maternity analyses could be performed. Although we provide revised estimates for these females here, for comparison with past estimates, the associated uncertainties may well be even larger than indicated. While we consider point estimates here for direct comparison to past results, the uncertainty in our estimates (e.g., Figure 8 and Figure S5) is inherent. We hope that providing age estimates in the form of probability distributions will contribute to better understanding and allow future studies to more readily incorporate uncertainty into statistical analysis.

Age estimates have informed several key findings related to RKW biology, in particular the identification of a prolonged post‐reproductive lifespan in females and the positive influence that mothers and grandmothers have on the survival of their progeny. These findings have made RKWs a focal species for understanding the evolution of menopause, a rare phenomenon among non‐human animals. Our results indicate that NRKW lifespans are likely somewhat shorter than previously estimated and imply that the post‐reproductive life phase may also be relatively shorter. While revisiting past results to ensure the accuracy of details seems wise, most of our modal age estimates do not differ greatly from past results (Figure 8). Further, our demographic analyses of known‐age individuals still suggest that reproductive senescence is decoupled from somatic senescence in NRKWs; fecundity decreases markedly after age 40 (Figure 2) while survivorship appears to remain reasonably high for much longer (Figure 4). Future analyses will draw on the combined data from known‐age and estimated‐age individuals to update our understanding of RKW senescence patterns.

Both NRKW and SRKW populations face considerable conservation threats, and understanding the long‐term implications of those threats requires an understanding of demographic patterns in the respective RKW populations. Inference about relevant population responses to stressors, mediated by effects on birth and death rates, relies on demographic data that are themselves informed by age estimates (Vélez‐Espino et al. 2014, 2015). Without accurate age information, inference about age‐specific rates of mortality and natality will be inaccurate, leading to potentially biased inference about relevant population responses.

Like the NRKW population, the SRKW population has been the subject of an annual photographic census since 1974 (Center for Whale Research 2016), enabling demographic comparison of the two populations. Most vital rates are similar, although SRKWs show reduced calf survival and reduced fecundity in older females (Vélez‐Espino et al. 2014). The core of our updated age estimation technique could thus be readily applied, with limited adjustments for population‐specific demographic rates, to the SRKW population. Unlike the NRKW population, however, which has seen an average 2.2% annual population increase since 1973 (DFO 2022), the SRKW population has stagnated and shows signs of decline (Center for Whale Research 2016; Murray et al. 2021). Care would be required to determine the appropriate population‐growth assumption, although the differences we observed here are relatively minor (Figure 6).

Our approach to age estimation, characterised by well‐understood probability relationships and age‐at‐event data, could be used to help guide age estimation for other species. While near‐complete annual census and morphological data will not be available in all cases, we have demonstrated how to incorporate uncertainty into our age estimates, and the techniques could be adapted to situations where additional uncertainty exists. In general, our approach requires information about two life history features: (1) the probability (or frequency) with which a recognisable event occurs at each age, and (2) the probability of an encountered individual being each relevant age. These two factors form the numerator of Bayes' theorem, as we used it to estimate age (e.g., Equation (2)), while the denominator is calculated via summation across a partition involving (1) and (2) (e.g., Equation (4)). Such information may sound simple to acquire, but in practice complications exist. One is that the age‐specific probabilities of the focal events may themselves be challenging to calculate. We saw this in the case of calculating the probability of a female having an oldest living offspring of a given age, which required combining information about maternal fecundity and juvenile survival rates. Another complication is the requirement of comprehensive (or what can be reasonably assumed to be comprehensive) information about occurrence of the relevant life history event throughout life, along with information about survival to the relevant ages. If the recognisable event is likely to happen too late in life, age estimation may become moot. Thus, our approach seems best suited to life history events occurring at relatively young ages (e.g., first reproduction, maturation, etc.). Our approach could also be adapted, for example, to formalise age estimation based on morphometric size‐at‐age data. As with any approach, care will be needed to adapt the method to new situations.

Other species or populations to which our methods could be adapted likely include many for which study duration—even if many years—is short relative to animal lifespan. Even for relatively longer studies, estimating the age of individuals present at the start of study may enable useful insight. Other populations of killer whales, for example Crozet Island (Tixier et al. 2017) and Marion Island (Jordaan et al. 2020) populations, in the Southern Ocean, present obvious potential applications. In these cases, methods almost identical to those used here might be applicable, or aspects of the relationships we have characterised might judiciously stand in for missing data. In other cases, as for age estimation using morphometric data in elephants (O'Connell‐Rodwell et al. 2022), adaptations of our approach that relate size or growth rate to age might be able to hone existing age estimates. In still other cases, further adaptation of our methods—notably involving the idea of discounting older ages—might be able to reduce uncertainty in physical ageing methods that rely on counting growth layers (e.g., in teeth or bone; Read et al. 2018).

We have shown that giving due consideration to age‐related discounting (i.e., the reduced chances of observing older individuals) can be useful when age‐at‐event data are used to infer unknown ages. In some cases, however, the natural paucity of older individuals in (near‐)random samples from a population may serve as an empirical, rather than mathematical, discounting for age. For example, a recent epigenetic ageing tool developed for common bottlenose dolphins ( *Tursiops truncatus* ) based age estimates on an initial sample that was biased—as required—towards young individuals (Beal et al. 2019). Here, we relied on demographic and probabilistic relationships to generate age estimates where we could not construct empirical (automatically discounted) age distributions for fixed events, because NRKW lifespans exceed the current study duration, although we could have adopted the latter approach in certain cases (e.g., age at “sprouting” for males).

Species‐specific techniques for ageing long‐lived or difficult‐to‐track individuals have continued to be developed (e.g., Cailliet et al. 2001; Shrader et al. 2005; Gabriele et al. 2010), and in multiple cases, effective age estimation has been made possible by long‐term studies (e.g., Fritz 2017; Gabriele et al. 2017; Mann and Karniski 2017). Furthermore, long‐term studies form the basis for much of our detailed understanding of ecology, animal behaviour, evolution, and conservation, and associated contributions to knowledge tend to increase over time (Clutton‐Brock and Sheldon 2010). Even in situations where studies are not long‐term from the perspective of a focal species, it may be possible to use one set of information to fill gaps, as we have done here, reducing what would otherwise be very large uncertainties. Maximising the information we can gain from long‐term studies is of great importance, since such studies are often fundamental to our understanding of population processes, itself necessary to understand how populations might respond to a changing world.

## Author Contributions

**Andrew W. Bateman:** conceptualization (lead), data curation (supporting), formal analysis (lead), funding acquisition (equal), methodology (lead), software (lead), visualization (lead), writing – original draft (lead), writing – review and editing (lead). **Jessica MacLean:** software (supporting), validation (equal), writing – original draft (supporting). **Eva Stredulinsky:** data curation (equal), investigation (supporting), software (supporting), validation (equal), writing – review and editing (equal). **Brianna Wright:** investigation (supporting), validation (equal), writing – review and editing (equal). **Graeme Ellis:** data curation (equal), investigation (equal), methodology (equal), project administration (equal), validation (equal), writing – review and editing (equal). **Thomas Doniol‐Valcroze:** data curation (equal), investigation (equal), project administration (equal), validation (equal), writing – review and editing (equal). **Chris Darimont:** funding acquisition (equal), resources (equal), supervision (lead), writing – review and editing (equal). **John K. B. Ford:** data curation (equal), funding acquisition (equal), investigation (lead), methodology (equal), project administration (equal), resources (equal), supervision (equal), validation (equal), writing – review and editing (equal).

## Conflicts of Interest

The authors declare no conflicts of interest.

## Supporting information


Appendix S1.

## Data Availability

The data that support the findings of this study are openly available in Dryad at https://doi.org/10.5061/dryad.g79cnp5xc.
