# Supplementary material for: Revised Age Estimates for Northern Resident Killer Whales (Orcinus orca) Based on Observed Life‐History Events and Demographic Discounting
Source: Ecol Evol. 2025 Mar 9;15(3):e70981. doi: 10.1002/ece3.70981 (PMC11890658; doi:10.1002/ece3.70981)
Supplement: Supplementary file 1 — Appendix S1. [file ECE3-15-e70981-s001.docx]

**Appendix S1: supplemental methods and results**

**Modification for females that produced their first known offspring during the study**

For females that entered the study at an unknown age and gave birth to their first known offspring in the *n*^th^ subsequent census observation, we can make Equation (2) conditional on *n*, with fixed offspring age *y* = 0 when the female’s first offspring was observed:

$\mathrm{P}(x|0\cap n)=\left.\frac{\mathrm{P}(y|x\cap n)\mathrm{P}(x|n)}{\mathrm{P}(y|n)}\right|_{y=0}$ . (S1)

To begin parameterising Equation (S1), we can modify Equation (3) to consider that no offspring were produced during the *n* years of observation before the female’s first observed calf. That is, we need not consider the possibility that, during those *n* years, the mother gave birth to offspring that subsequently died, given that the pregnancies or offspring would have been observed:

$\left.\mathrm{P}(y|x\cap n)\right|_{y=0}=\left[\prod_{t=0}^{x-n-1} \left[1-R(t)S(x-t)\right]\prod_{t=x-n}^{x-1} \left[1-R(t)\right]\right]R(x)$. (S2)

P(*x*|*n*) is simply the age distribution, accounting for population growth, truncated so that *x*≥*n* and rescaled, since we know the mother to be at least *n* years old, having observed her for that much time. Note that here we are not presupposing the mother to be of a certain age when she was first seen, simply putting an extreme minimum bound on her age at the time she has her first observed calf. Similar to Equation (4), P(*y*|*n*)|*_y_*_=0_ becomes:

$\left.\mathrm{P}(y|n)\right|_{y=0}=\sum_{x=n}^{\infty} \left[\left.\mathrm{P}(y|x\cap n)\right|_{y=0}\mathrm{P}(x|n)\right]$. (S3)

**Modification for males that “sprouted” during the study**

Building on Equations (S1) through (S3), we consider age estimation for males that exhibited the onset of sexually dimorphic dorsal fin growth (“sprouting”) after *n* years in the study. We denote the event that a male first sprouts as *FS*, so that:

$\mathrm{P}_{m}(x|FS\cap n)=\left.\frac{\mathrm{P}_{m}(FS|x\cap n)\mathrm{P}_{m}(x|n)}{\mathrm{P}_{m}(FS|n)}\right|_{y=0}$ . (S4)

The probability that a male first sprouts at age *x* (≥*n*), P*_m_*(*FS*|*x∩n*), is:

$$\mathrm{P}_{m}(FS|x\cap n)=\left[\prod_{t=0}^{x-n-1} \left[1-F(t)\right]\prod_{t=x-n}^{x-1} \left[1-F(t)\right]\right]F(x)$$

$=\left[\prod_{t=0}^{x-1} \left[1-F(t)\right]\right]F(x)$, (S5)

where *F*(*t*) is the probability that a male has first sprouted at age *t* (i.e. between census in year *t*-1 and census in year *t*). To estimate *F*(*t*), we used the same approach as used to estimate annual mortality, with the assumption that the “hazard” of sprouting, *h­_S_*(*t*), is zero until age nine (by which point no males have been observed to sprout), and increases linearly thereafter. In parameterising this model, we fit the function describing the probability that a male did not sprout in a given year, 1*-F*(*t*) = exp($-\int_{t-1}^{t} h_{S}(\tau)d\tau$), to corresponding data (Figure S1).


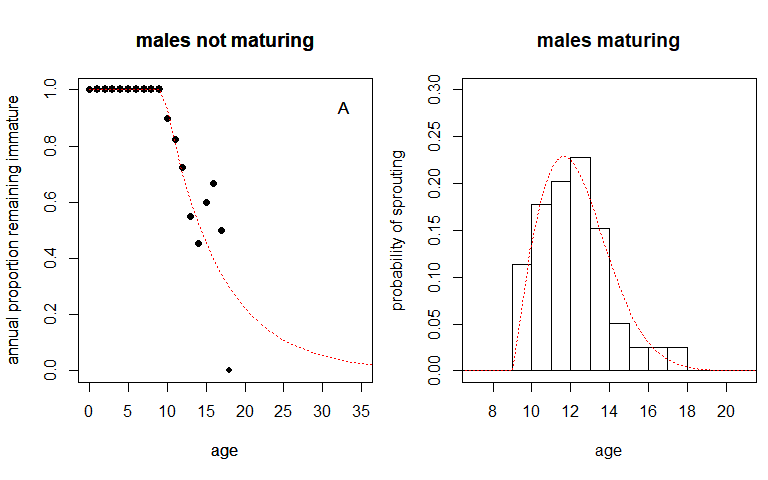


Figure S1: (A) Observed (points, area proportional to sample size) and estimated (red dashed curve) annual probability that an immature NRKW male has yet to “sprout” (exhibit the onset of sexually dimorphic dorsal fin growth characteristic of maturation). (B) Distribution of sprouting age observed (bars) and estimated (red dashed curve) from the relationship estimated for (A).

P*_m_*(*x*|*n*) is the male age distribution, accounting for population growth, truncated so that *x*≥*n* and rescaled to sum to one. Again, we estimated a bathtub-shaped hazard function by fitting the corresponding annual survivorship to data for males of known age (Figure S2A). From this, we could estimate the cumulative survivorship curve (Figure S2B). As with females, we imputed the hazard function to age fifteen with that for all individuals, to avoid survivorship bias in estimating P*_m_*(*x*|*n*).


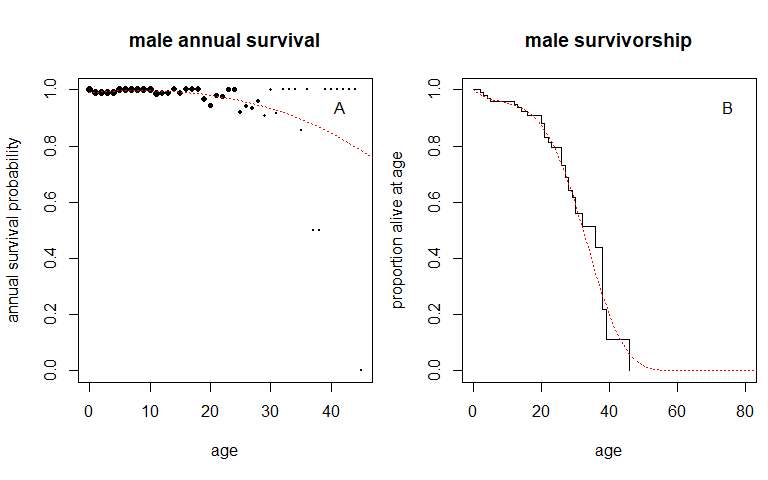


Figure S2: Annual survival (A) observed (points, area proportional to sample size) and estimated (red dashed curve), and survivorship (B) observed (black) and estimated (red dashed) for male NRKWs of known age. Note low early-life mortality in B, relative to Figure 1B, due to survivorship estimation bias (individuals that die before being sexed are absent from this analysis).

Similar to previous cases, P(*FS*|*n*) is calculated by partitioning by age, *x*:

$\mathrm{P}(FS|n)=\sum_{x=n}^{\infty} \left[\mathrm{P}(FS|x\cap n)\mathrm{P}(x|n)\right]$. (S6)

**Modification for males that attained full maturity during the study**

For males that had already begun their dorsal fin growth at the start of the study, and which later displayed a full-size (dorsal) fin (FSF) after *n* years in the study, we can modify Equation (S4):

$\mathrm{P}_{m}(x|FSF_{0}\cap n)=\left.\frac{\mathrm{P}_{m}(FSF_{0}|x\cap n)\mathrm{P}_{m}(x|n)}{\mathrm{P}_{m}(FSF_{0}|n)}\right|_{y=0}$ , (S7)

where P*_m_*(*FSF*_0_) is the probability that a male first achieves a full-sized fin. The components of Equation (S7) are calculated similarly to the corresponding components of Equation (S4). Here, for simplicity, we did not take into account the information that these males had already begun their dorsal fin growth, and were therefore older than *n* years. Figure S3 compares P*_m_*(*FS*|*x∩n*) and P*_m_*(*FSF*_0_|*x∩n*) for *n*=0.


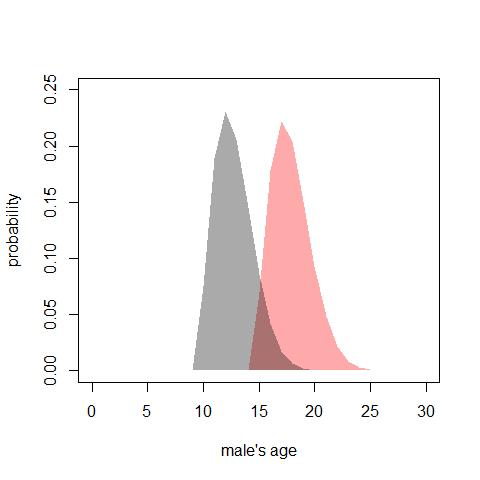


Figure S3: Probability distributions for a male NRKW’s age, given the male began dorsal fin growth during the study (grey distribution) or had already begun dorsal fin growth when first observed and later achieved asymptotic dorsal fin size (red distribution). Distributions assume a NRKW population growth rate of 2.2%.

**Modification for males that entered the study already mature**

For males that entered the study with a full-size dorsal fin, Equation (S7) becomes:

$\mathrm{P}_{m}(x|FSF)=\left.\frac{\mathrm{P}_{m}(FSF|x)\mathrm{P}_{m}(x)}{\mathrm{P}_{m}(FSF)}\right|_{y=0}$ , (S8)

where P*_m_*(*FSF*) is the probability that a male has a full-sized fin. P*_m_*(*FSF*|*x*) is simply the cumulative distribution function corresponding to the P*_m_*(*FSF*_0_|*x*) probability mass function (i.e. the probability that males of age *x* had first achieved a full-sized fin in that year or any year prior). Note that Equation (S8) is not dependent on the male having survived for a given time, because it deals with the state of males when they were first seen. Without the information on when a focal male matured, the age-estimate uncertainty becomes much greater (Figure S4) than for estimates based on known maturation dates (Figure S3).


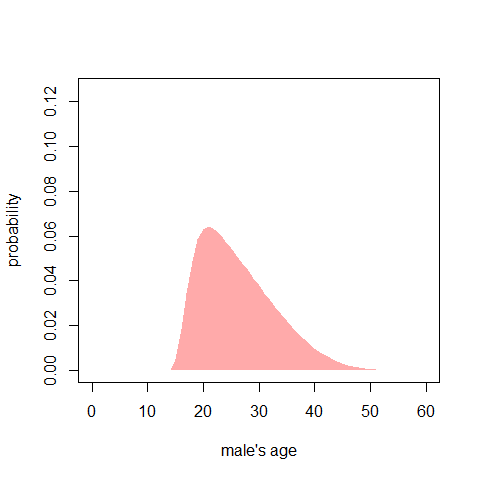


Figure S4: Probability distributions for a male NRKW’s age, given the male had achieved asymptotic dorsal fin size before he was first seen. Distribution assumes a NRKW population growth rate of 2.2%.

**Final estimated year-of-birth distributions**

The final year-of-birth distributions are shown in Figure S5, with summary statistics provided in Table S1. Best-fit lognormal parameters are provided in Table S2, with the associated approximations illustrated in Figures S6 and S7.


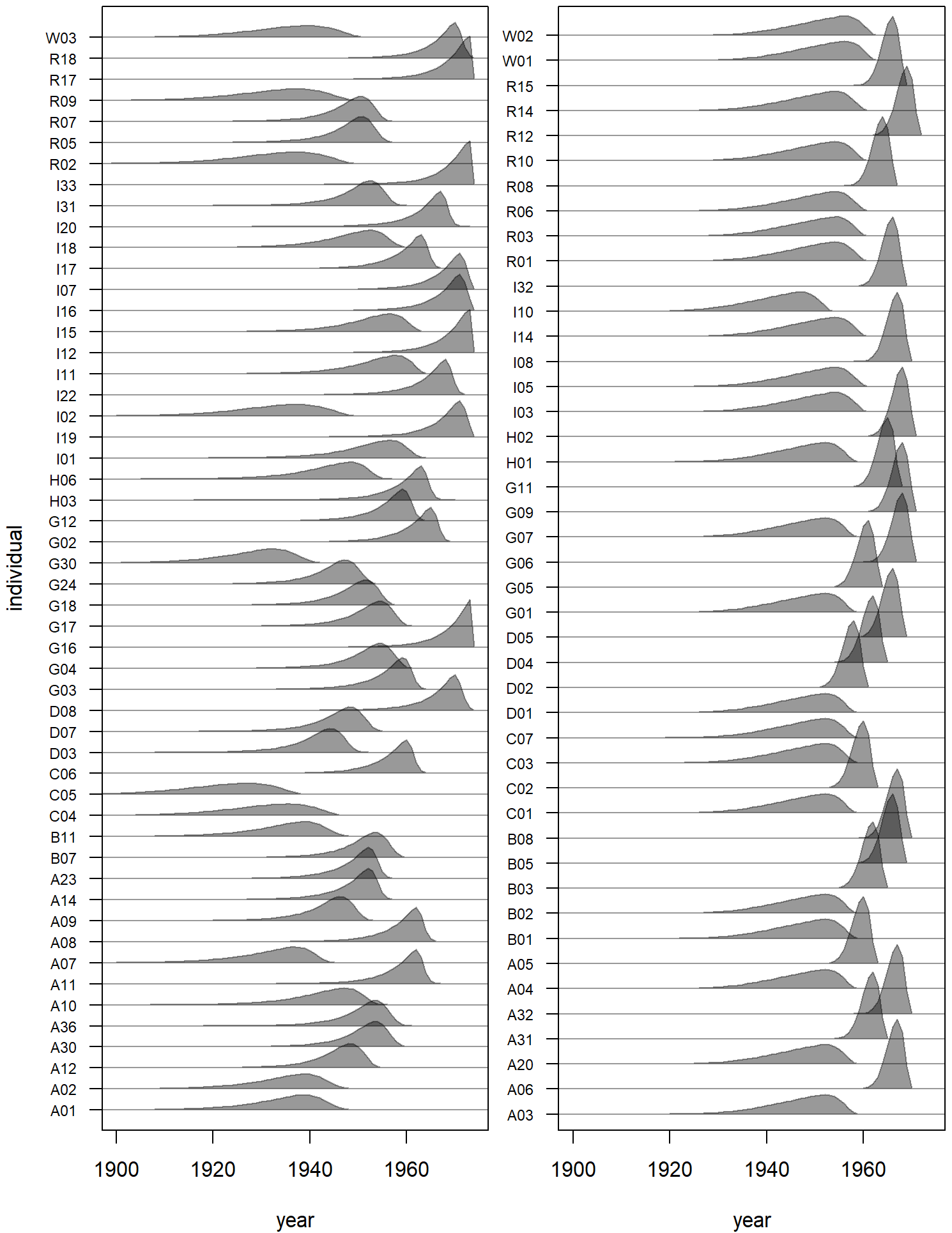


Figure S5: Year-of-birth estimates for NRKW females (left panel, n=52) and males (right panel, n=44), for which age could not be directly estimated. For females, age estimates relied on offspring age (itself uncertain) or year of first reproduction. For males, age estimates relied on year of maturation or, if the male was mature when first seen, the distribution of mature-male age.

Table S1: Summary statistics for year-of-birth (YoB) distributions estimated for NRKWs. Ageing methods: OLO = oldest living offspring, FSF = full size fin, FS = first sprout (initial dorsal fin growth), FM = first mature, FC = first calf (see text for clarification of methods)

| individual | sex | YoB mode | YoB mean | YoB 2.5%ile | YoB median | YoB 97.5%ile | method |
| --- | --- | --- | --- | --- | --- | --- | --- |
| A01 | F | 1939 | 1933.766 | 1913 | 1936 | 1945 | OLO |
| A02 | F | 1939 | 1933.766 | 1913 | 1936 | 1945 | OLO |
| A03 | M | 1952 | 1946.787 | 1931 | 1948 | 1956 | FSF |
| A06 | M | 1967 | 1966.275 | 1962 | 1966 | 1969 | FS |
| A12 | F | 1948 | 1945.058 | 1930 | 1946 | 1952 | OLO |
| A20 | M | 1952 | 1946.787 | 1931 | 1948 | 1956 | FSF |
| A30 | F | 1954 | 1950.355 | 1936 | 1952 | 1958 | OLO |
| A31 | M | 1962 | 1961.151 | 1957 | 1961 | 1964 | FM |
| A32 | M | 1967 | 1966.275 | 1962 | 1966 | 1969 | FS |
| A36 | F | 1954 | 1950.355 | 1936 | 1952 | 1958 | OLO |
| A04 | M | 1952 | 1946.787 | 1931 | 1948 | 1956 | FSF |
| A10 | F | 1947 | 1942.085 | 1922 | 1944 | 1952 | OLO |
| A11 | F | 1962 | 1958.306 | 1944 | 1960 | 1964 | FC |
| A05 | M | 1960 | 1959.151 | 1955 | 1959 | 1962 | FM |
| A07 | F | 1937 | 1931.303 | 1910 | 1933 | 1942 | OLO |
| A08 | F | 1962 | 1958.306 | 1944 | 1960 | 1964 | FC |
| A09 | F | 1946 | 1942.979 | 1927 | 1944 | 1950 | OLO |
| A14 | F | 1952 | 1948.529 | 1934 | 1950 | 1955 | OLO |
| A23 | F | 1952 | 1948.528 | 1934 | 1950 | 1955 | OLO |
| B01 | M | 1952 | 1946.787 | 1931 | 1948 | 1956 | FSF |
| B02 | M | 1952 | 1946.787 | 1931 | 1948 | 1956 | FSF |
| B03 | M | 1962 | 1961.151 | 1957 | 1961 | 1964 | FM |
| B05 | M | 1966 | 1965.275 | 1961 | 1965 | 1968 | FS |
| B07 | F | 1954 | 1950.355 | 1936 | 1952 | 1958 | OLO |
| B08 | M | 1967 | 1966.275 | 1962 | 1966 | 1969 | FS |
| B11 | F | 1939 | 1933.766 | 1913 | 1936 | 1945 | OLO |
| C01 | M | 1952 | 1946.787 | 1931 | 1948 | 1956 | FSF |
| C02 | M | 1960 | 1959.151 | 1955 | 1959 | 1962 | FM |
| C03 | M | 1952 | 1946.787 | 1931 | 1948 | 1956 | FSF |
| C04 | F | 1935 | 1930.015 | 1908 | 1932 | 1944 | OLO |
| C05 | F | 1927 | 1921.626 | 1899 | 1923 | 1936 | OLO |
| C06 | F | 1960 | 1956.243 | 1942 | 1958 | 1962 | OLO |
| C07 | M | 1952 | 1946.787 | 1931 | 1948 | 1956 | FSF |
| D01 | M | 1952 | 1946.787 | 1931 | 1948 | 1956 | FSF |
| D02 | M | 1958 | 1957.151 | 1953 | 1957 | 1960 | FM |
| D03 | F | 1944 | 1940.895 | 1925 | 1942 | 1948 | OLO |
| D04 | M | 1962 | 1961.151 | 1957 | 1961 | 1964 | FM |
| D05 | M | 1966 | 1965.275 | 1961 | 1965 | 1968 | FS |
| D07 | F | 1948 | 1945.058 | 1930 | 1946 | 1952 | OLO |
| D08 | F | 1970 | 1966.518 | 1953 | 1968 | 1972 | FC |
| G01 | M | 1952 | 1946.787 | 1931 | 1948 | 1956 | FSF |
| G03 | F | 1959 | 1955.75 | 1942 | 1957 | 1962 | OLO |
| G04 | F | 1955 | 1951.386 | 1937 | 1953 | 1959 | OLO |
| G05 | M | 1961 | 1960.151 | 1956 | 1960 | 1963 | FM |
| G06 | M | 1968 | 1967.275 | 1963 | 1967 | 1970 | FS |
| G07 | M | 1952 | 1946.787 | 1931 | 1948 | 1956 | FSF |
| G09 | M | 1968 | 1967.275 | 1963 | 1967 | 1970 | FS |
| G11 | M | 1965 | 1964.275 | 1960 | 1964 | 1967 | FS |
| G16 | F | 1973 | 1968.729 | 1955 | 1970 | 1973 | FC |
| G17 | F | 1955 | 1951.386 | 1937 | 1953 | 1959 | OLO |
| G18 | F | 1952 | 1948.292 | 1933 | 1950 | 1956 | OLO |
| G24 | F | 1947 | 1944.019 | 1929 | 1945 | 1951 | OLO |
| G30 | F | 1932 | 1927.109 | 1905 | 1929 | 1939 | OLO |
| G02 | F | 1965 | 1961.367 | 1947 | 1963 | 1967 | FC |
| G12 | F | 1959 | 1955.75 | 1942 | 1957 | 1962 | OLO |
| H01 | M | 1952 | 1946.787 | 1931 | 1948 | 1956 | FSF |
| H02 | M | 1968 | 1967.275 | 1963 | 1967 | 1970 | FS |
| H03 | F | 1963 | 1959.319 | 1945 | 1961 | 1965 | FC |
| H06 | F | 1948 | 1943.138 | 1923 | 1945 | 1953 | OLO |
| I01 | F | 1956 | 1951.581 | 1933 | 1953 | 1961 | OLO |
| I03 | M | 1954 | 1948.787 | 1933 | 1950 | 1958 | FSF |
| I19 | F | 1971 | 1967.472 | 1954 | 1969 | 1973 | FC |
| I02 | F | 1937 | 1932.115 | 1910 | 1934 | 1946 | OLO |
| I05 | M | 1954 | 1948.787 | 1933 | 1950 | 1958 | FSF |
| I08 | M | 1967 | 1966.275 | 1962 | 1966 | 1969 | FS |
| I14 | M | 1954 | 1948.787 | 1933 | 1950 | 1958 | FSF |
| I22 | F | 1968 | 1964.471 | 1951 | 1966 | 1970 | FC |
| I10 | M | 1947 | 1941.787 | 1926 | 1943 | 1951 | FSF |
| I11 | F | 1958 | 1952.762 | 1934 | 1954 | 1962 | OLO |
| I12 | F | 1973 | 1968.614 | 1955 | 1970 | 1973 | FC |
| I15 | F | 1956 | 1951.581 | 1933 | 1953 | 1961 | OLO |
| I16 | F | 1971 | 1967.472 | 1954 | 1969 | 1973 | FC |
| I07 | F | 1971 | 1967.472 | 1954 | 1969 | 1973 | FC |
| I17 | F | 1963 | 1959.319 | 1945 | 1961 | 1965 | FC |
| I18 | F | 1952 | 1947.408 | 1928 | 1949 | 1957 | OLO |
| I20 | F | 1967 | 1963.435 | 1950 | 1965 | 1969 | FC |
| I31 | F | 1953 | 1949.324 | 1934 | 1951 | 1957 | OLO |
| I32 | M | 1966 | 1965.275 | 1961 | 1965 | 1968 | FS |
| I33 | F | 1973 | 1968.614 | 1955 | 1970 | 1973 | FC |
| R01 | M | 1954 | 1948.787 | 1933 | 1950 | 1958 | FSF |
| R02 | F | 1937 | 1932.115 | 1910 | 1934 | 1946 | OLO |
| R03 | M | 1954 | 1948.787 | 1933 | 1950 | 1958 | FSF |
| R05 | F | 1951 | 1947.491 | 1932 | 1949 | 1955 | OLO |
| R06 | M | 1954 | 1948.787 | 1933 | 1950 | 1958 | FSF |
| R07 | F | 1950 | 1947.258 | 1932 | 1949 | 1955 | OLO |
| R08 | M | 1964 | 1963.275 | 1959 | 1963 | 1966 | FS |
| R09 | F | 1937 | 1932.115 | 1910 | 1934 | 1946 | OLO |
| R10 | M | 1954 | 1948.787 | 1933 | 1950 | 1958 | FSF |
| R12 | M | 1969 | 1968.275 | 1964 | 1968 | 1971 | FS |
| R14 | M | 1954 | 1948.787 | 1933 | 1950 | 1958 | FSF |
| R15 | M | 1966 | 1965.275 | 1961 | 1965 | 1968 | FS |
| R17 | F | 1973 | 1968.614 | 1955 | 1970 | 1973 | FC |
| R18 | F | 1970 | 1966.518 | 1953 | 1968 | 1972 | FC |
| W01 | M | 1956 | 1950.787 | 1935 | 1952 | 1960 | FSF |
| W02 | M | 1956 | 1950.787 | 1935 | 1952 | 1960 | FSF |
| W03 | F | 1939 | 1934.213 | 1912 | 1936 | 1948 | OLO |

Table S2: least-squares parameter estimates for discretised lognormal approximations of empirically calculated age distributions (see supplemental code). The discretisation takes the form: P(birth occurred n years before the zero year) = $\int_{n}^{n+1} f\left( x \right)\mathrm{dx}\boldsymbol{,}$ where *f*(x) is a lognormal random variable with specified mean and standard deviation on the natural log scale. Note: for completeness, this table contains lognormal parameters for individuals whose age was estimated directly (i.e. not using one of the methods referenced in Table S1; see text for details)

| individual | zero year | mean (log scale) | standard deviation (log scale) |
| --- | --- | --- | --- |
| A01 | 1950 | 2.714201 | 0.50215 |
| A02 | 1950 | 2.714201 | 0.50215 |
| A03 | 1958 | 2.377499 | 0.716213 |
| A06 | 1969 | 1.149463 | 0.602265 |
| A12 | 1956 | 2.309566 | 0.461584 |
| A20 | 1958 | 2.377499 | 0.716213 |
| A30 | 1961 | 2.285355 | 0.457826 |
| A31 | 1964 | 1.187031 | 0.602082 |
| A32 | 1969 | 1.149463 | 0.602265 |
| A33 | 1973 | 0.675946 | 0.23765 |
| A36 | 1961 | 2.285355 | 0.457826 |
| A38 | 1973 | 0.906253 | 0.100565 |
| A04 | 1958 | 2.377499 | 0.716213 |
| A10 | 1957 | 2.613735 | 0.497231 |
| A11 | 1968 | 2.145596 | 0.412159 |
| A24 | 1973 | 1.790017 | 0.082796 |
| A05 | 1962 | 1.187031 | 0.602082 |
| A07 | 1947 | 2.661324 | 0.511572 |
| A08 | 1968 | 2.145596 | 0.412159 |
| A09 | 1954 | 2.314287 | 0.464198 |
| A14 | 1958 | 2.121136 | 0.461752 |
| A21 | 1973 | 1.790017 | 0.082796 |
| A23 | 1958 | 2.121271 | 0.461655 |
| A25 | 1973 | 0.376785 | 0.169504 |
| A26 | 1973 | 0.376785 | 0.169504 |
| A27 | 1973 | 0.376785 | 0.169504 |
| B01 | 1958 | 2.377499 | 0.716213 |
| B02 | 1958 | 2.377499 | 0.716213 |
| B03 | 1964 | 1.187031 | 0.602082 |
| B04 | 1973 | 2.296723 | 0.150793 |
| B05 | 1968 | 1.149463 | 0.602265 |
| B07 | 1961 | 2.285355 | 0.457826 |
| B08 | 1969 | 1.149463 | 0.602265 |
| B11 | 1950 | 2.714201 | 0.50215 |
| C01 | 1958 | 2.377499 | 0.716213 |
| C02 | 1962 | 1.187031 | 0.602082 |
| C03 | 1958 | 2.377499 | 0.716213 |
| C04 | 1949 | 2.905415 | 0.534918 |
| C05 | 1941 | 2.929342 | 0.536323 |
| C06 | 1966 | 2.153505 | 0.41418 |
| C07 | 1958 | 2.377499 | 0.716213 |
| C09 | 1973 | 0.376785 | 0.169504 |
| C10 | 1973 | 0.376785 | 0.169504 |
| D01 | 1958 | 2.377499 | 0.716213 |
| D02 | 1960 | 1.187031 | 0.602082 |
| D03 | 1952 | 2.319552 | 0.46706 |
| D04 | 1964 | 1.187031 | 0.602082 |
| D05 | 1968 | 1.149463 | 0.602265 |
| D07 | 1956 | 2.309566 | 0.461584 |
| D08 | 1973 | 1.716043 | 0.653807 |
| D09 | 1973 | 0.376785 | 0.169504 |
| G01 | 1958 | 2.377499 | 0.716213 |
| G03 | 1965 | 2.103854 | 0.454498 |
| G04 | 1962 | 2.283493 | 0.456993 |
| G05 | 1963 | 1.187031 | 0.602082 |
| G06 | 1970 | 1.149463 | 0.602265 |
| G07 | 1958 | 2.377499 | 0.716213 |
| G09 | 1970 | 1.149463 | 0.602265 |
| G11 | 1967 | 1.149463 | 0.602265 |
| G16 | 1973 | 1.1772 | 1.287283 |
| G17 | 1962 | 2.283493 | 0.456993 |
| G18 | 1959 | 2.289132 | 0.459834 |
| G20 | 1973 | 0.675946 | 0.23765 |
| G24 | 1955 | 2.311884 | 0.462826 |
| G26 | 1973 | 0.906253 | 0.100565 |
| G29 | 1973 | 1.247631 | 0.071642 |
| G30 | 1944 | 2.753871 | 0.508296 |
| G02 | 1971 | 2.136627 | 0.40911 |
| G08 | 1973 | 0.675946 | 0.23765 |
| G12 | 1965 | 2.103854 | 0.454498 |
| H01 | 1958 | 2.377499 | 0.716213 |
| H02 | 1970 | 1.149463 | 0.602265 |
| H03 | 1969 | 2.143608 | 0.411959 |
| H06 | 1958 | 2.610414 | 0.49662 |
| I01 | 1965 | 2.505681 | 0.52594 |
| I03 | 1960 | 2.377499 | 0.716213 |
| I19 | 1973 | 1.546505 | 0.836632 |
| I02 | 1951 | 2.899879 | 0.534214 |
| I05 | 1960 | 2.377499 | 0.716213 |
| I08 | 1969 | 1.149463 | 0.602265 |
| I14 | 1960 | 2.377499 | 0.716213 |
| I22 | 1973 | 2.005991 | 0.461612 |
| I10 | 1953 | 2.377499 | 0.716213 |
| I11 | 1966 | 2.484576 | 0.52208 |
| I12 | 1973 | 1.221017 | 1.239474 |
| I15 | 1965 | 2.505681 | 0.52594 |
| I16 | 1973 | 1.546505 | 0.836632 |
| I07 | 1973 | 1.546505 | 0.836632 |
| I17 | 1969 | 2.143608 | 0.411959 |
| I18 | 1962 | 2.592242 | 0.492342 |
| I20 | 1973 | 2.129097 | 0.40418 |
| I31 | 1960 | 2.287196 | 0.458788 |
| I32 | 1968 | 1.149463 | 0.602265 |
| I33 | 1973 | 1.221017 | 1.239474 |
| R01 | 1960 | 2.377499 | 0.716213 |
| R02 | 1951 | 2.899879 | 0.534214 |
| R03 | 1960 | 2.377499 | 0.716213 |
| R04 | 1973 | 2.296723 | 0.150793 |
| R05 | 1959 | 2.358737 | 0.408402 |
| R06 | 1960 | 2.377499 | 0.716213 |
| R07 | 1958 | 2.291121 | 0.460929 |
| R08 | 1966 | 1.149463 | 0.602265 |
| R09 | 1951 | 2.899879 | 0.534214 |
| R10 | 1960 | 2.377499 | 0.716213 |
| R12 | 1971 | 1.149463 | 0.602265 |
| R14 | 1960 | 2.377499 | 0.716213 |
| R15 | 1968 | 1.149463 | 0.602265 |
| R17 | 1973 | 1.221017 | 1.239474 |
| R18 | 1973 | 1.716043 | 0.653807 |
| W01 | 1962 | 2.377499 | 0.716213 |
| W02 | 1962 | 2.377499 | 0.716213 |
| W03 | 1953 | 2.894513 | 0.533093 |


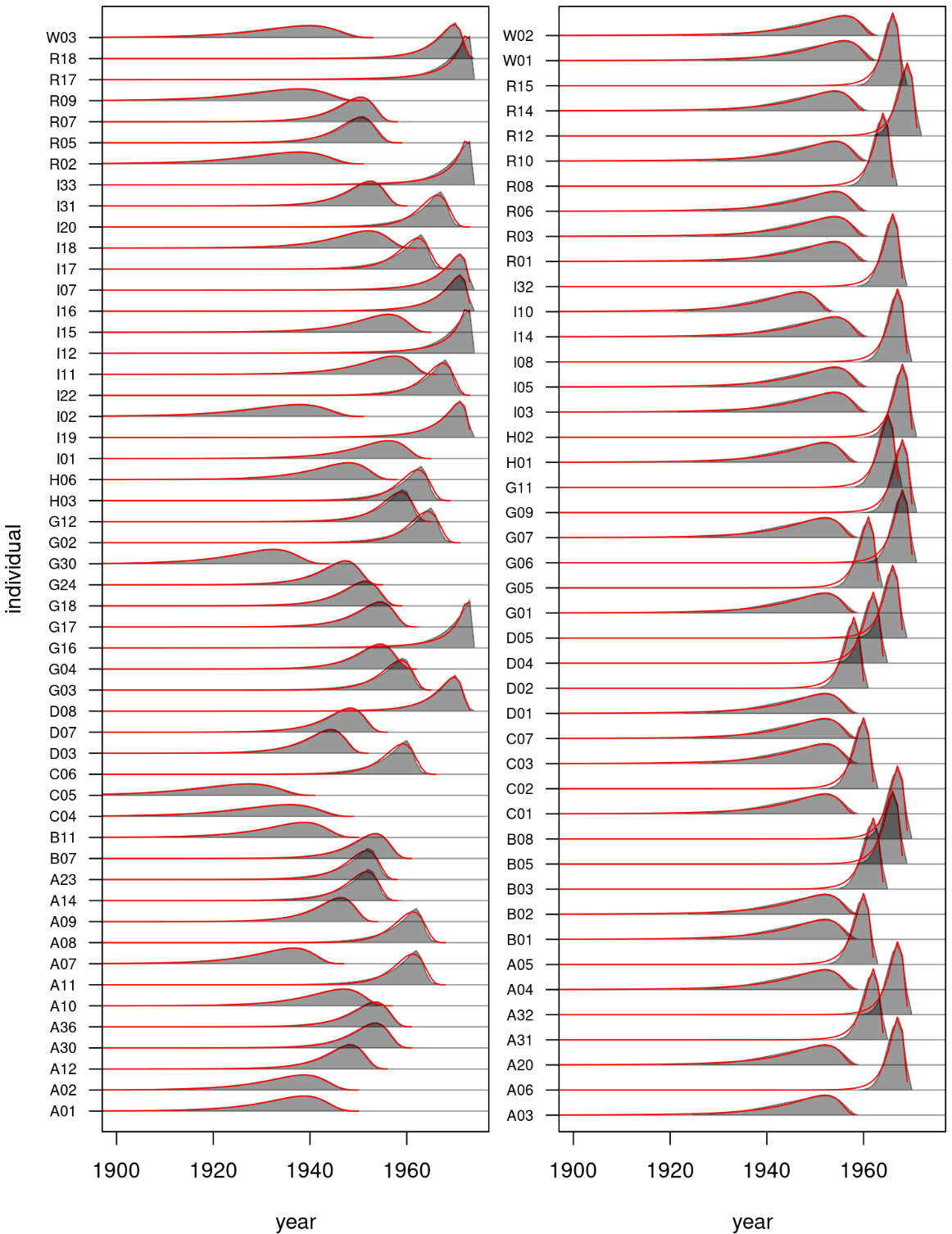


Figure S6: Year-of-birth estimate distributions (grey) for NRKW females (left panel, n=52) and males (right panel, n=44), for which age could not be directly estimated. Red lines represent least-squares approximations of the underlying distributions using discretised lognormal distributions.


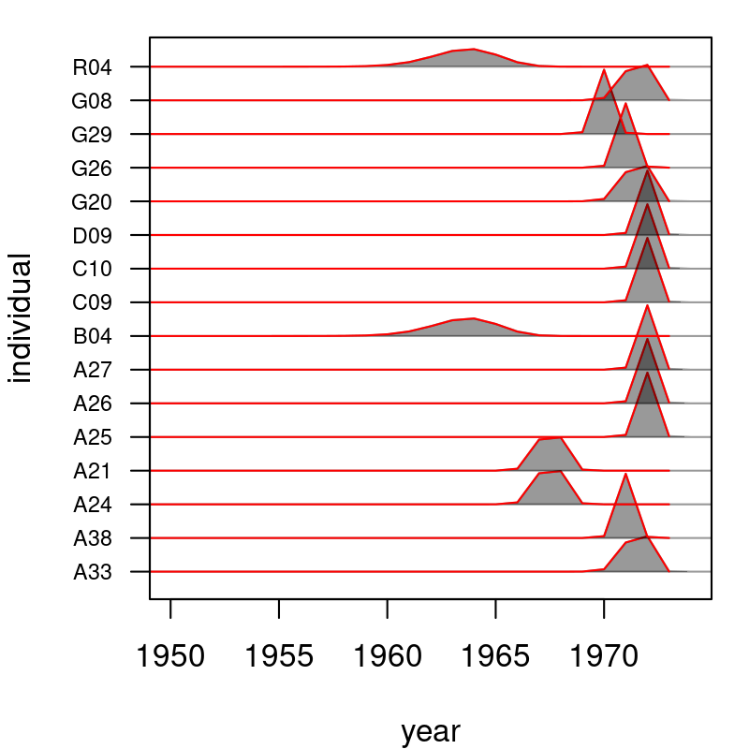


Figure S7: Year-of-birth estimate distributions (grey) for NRKWs that were very young when first observed, such that age could be directly estimated. Red lines represent least-squares approximations of the underlying distributions using discretised lognormal distributions. See text for details.
